# Batteries for wearables

**DOI:** 10.1093/nsr/nwac062

**Published:** 2022-03-31

**Authors:** Lie Wang, Ye Zhang, Peter G Bruce

**Affiliations:** National Laboratory of Solid State Microstructures, Jiangsu Key Laboratory of Artificial Functional Materials, Chemistry and Biomedicine Innovation Center (ChemBIC), Collaborative Innovation Center of Advanced Microstructures, College of Engineering and Applied Sciences, Nanjing University, China; National Laboratory of Solid State Microstructures, Jiangsu Key Laboratory of Artificial Functional Materials, Chemistry and Biomedicine Innovation Center (ChemBIC), Collaborative Innovation Center of Advanced Microstructures, College of Engineering and Applied Sciences, Nanjing University, China; Departments of Materials and Chemistry, University of Oxford, UK

## Abstract

This perspective article highlights the recent advances and future challenges of battery technologies for wearables.

Batteries that convert the stored chemical energy directly into electricity have been known for hundreds of years. Take the lithium-ion battery as representative because of its high energy-conversion efficiency and long cycle life. It is widely used as a power accessory for electronic devices and has become an important part of modern electronics. Despite its past successes, battery technology has progressed much more slowly compared to other electronic devices. This makes it appear to be powerless when facing emerging electronic technologies, especially wearable electronics, and has even become a bottleneck, hindering the development of this field [1]. Therefore, it is urgent to accelerate the innovation of battery technology. From this perspective, we highlight the main requirements and challenges of batteries for wearables (Fig. 1).

**Figure 1.**
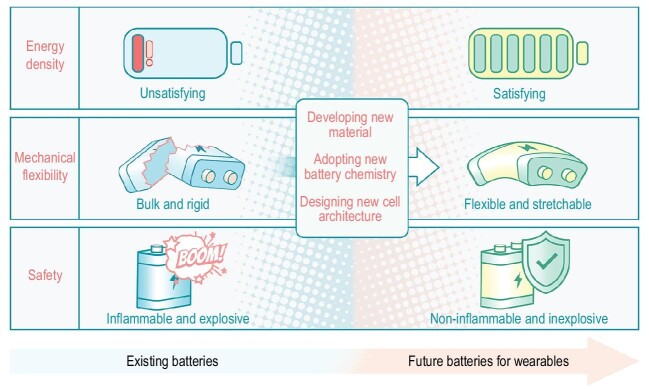
The pathway towards future batteries for wearables from the perspectives of energy density, mechanical flexibility and safety.

Battery life is one of the most concerning indicators of wearables and it is mainly determined by the energy density of the battery. As weight and volume in wearables are limited, batteries with higher energy densities can enable devices to run for longer periods. Lithium-ion batteries have some of the highest energy densities (100–265 Wh·kg^−1^ and 250–693 Wh·L^−1^) existing in battery technology but they still cannot meet the requirements of the wearable market. In particular, with the requirement for miniaturization and light weight of wearables, the space that a battery can occupy in the whole device is increasingly limited. Therefore, it is urgent to improve the energy density of batteries. The innovation of key battery materials with better performances is a feasible approach, including regulating the composition and morphology of electrode materials and developing new electrolytes [2]. However, as the energy densities of lithium-ion batteries head toward a saturation limit, the results obtained by this strategy will eventually reach a bottleneck. Thus, the adoption of new battery chemistry (such as metal–air batteries) with higher energy densities has been explored, which are considered as the next-generation batteries (with energy densities of >350 Wh·kg^−1^ and 750 Wh·L^−1^) to give electronics a longer battery life [3,4].

Wearable electronics are also expected to be flexible and even stretchable so that they can better fit our bodies while maintaining good wear comfort. To this end, battery technologies that robustly match the mechanical flexibility of the overall electronic system are required. However, existing batteries share prismatic or cylindrical shapes with rigid encapsulations, which are difficult to use in flexible and wearable electronics. Battery components such as active materials and current collectors are also intrinsically rigid and they would peel away from each other during deformation, resulting in deterioration of battery performance or even failure. To overcome these problems, there are two main strategies for the development of flexible batteries: improvement in mechanical flexibility among battery components by synthesizing new materials and designing flexible cell architectures [5]. For the first strategy, researchers have explored carbon-based materials (such as carbon textile, carbon nanotube film and graphene paper) that are highly flexible and have superior electrical conductivities to replace rigid current collectors or serve as freestanding electrodes [6]. For the second strategy, various flexible cell architectures, including film and fiber, have been verified to accommodate severe deformation states while maintaining the electrochemical performance of batteries [7].

Safety is another important factor in battery design for wearables since battery failure may lead to serious consequences. Over the past few years, accidents related to fires and explosions of batteries have frequently occurred worldwide, which have left enough lessons for people. The battery-explosion accident mainly originates from the thermal runaway caused by mechanical abuse (crash, penetration, etc.), electrochemical abuse (short-circuit, over-charge/discharge, etc.) and thermal abuse (overheat, thermal shock, fire, etc.). This safety hazard may be further increased when the battery is used in flexible wearable electronics, as these devices are subjected to more frequent stresses or deformations during use, increasing the possibility of mechanical damage to the battery. To minimize the safety risk, equipping battery-management systems or safety features such as fuses and current-interrupt devices with batteries represents a mature method [8]. However, these additional components take up more device space. Improvement of battery materials with better safety may be a more promising strategy. For instance, the construction of a protective coating in electrode materials and the addition of flame-retardant additives in electrolytes has proven effective in suppressing the occurrence of battery thermal runaway [9]. In addition, the development of novel batteries with high safety, such as aqueous batteries, has also been explored [10].

Although researchers have made significant progress in improving the energy densities of batteries, mechanical flexibility and safety, how to balance all aspects of battery performance will be a bigger challenge in the future. For example, batteries with high flexibility or safety tend to have low energy densities, while high-energy-density batteries lack mechanical flexibility and safety. In the short term, a single battery technology to outperform others in all three aspects may be difficult. Therefore, trade-offs need to be made to balance these performances according to the requirements of specific applications. Taking electronic textiles as an example, the requirement of battery energy density can be appropriately reduced on the premise of ensuring flexibility and safety. On the other hand, in the long term, efforts are still needed to develop new materials to continuously improve the overall performance of the battery, especially in developing new encapsulation materials with high flexibility, even stretchability, and low water/gas permeability, which is important for the development of high-performance, flexible and safe batteries for wearables.

In addition, how to integrate batteries into wearables is also a crucial issue for the future. Wearables such as electronic skins and electronic textiles are intrinsically soft with a finer structure so the traditional integration technology of rigid electronic devices may not be suitable for them. To this end, it is necessary to develop new integration methods between batteries and wearables to achieve systematic functional applications while easing the installation. In particular, for the booming smart textiles, it is more urgent to explore general and effective integration strategies to assemble fiber batteries into wearable systems. Fortunately, traditional textile technologies such as weaving have been proposed and demonstrated as an effective strategy [7]. Improvements to these textile technologies may further improve the efficiency of integration.

The development of new battery technologies requires the joint efforts of researchers in academy and industry, as well as multidisciplinary collaboration in different fields, including chemistry, physics, material science and engineering, energy science, electrical engineering and mechatronics engineering. It is expected that the emergence of these new technologies will offer countless opportunities to improve our future lives.
